# Portrait of a killer: Uncovering resistance mechanisms and global spread of *Acinetobacter baumannii*

**DOI:** 10.1371/journal.ppat.1011520

**Published:** 2023-08-10

**Authors:** Amy K. Cain, Mehrad Hamidian

**Affiliations:** 1 ARC Centre of Excellence in Synthetic Biology, School of Natural Sciences, Macquarie University, Sydney, NSW, Australia; 2 Australian Institute of Microbiology and Infection, University of Technology Sydney, Ultimo, NSW, Australia; Duke University School of Medicine, UNITED STATES

## Abstract

Antibiotic resistance is a growing global concern in the field of medicine as it renders bacterial infections difficult to treat and often more severe. *Acinetobacter baumannii* is a gram-negative bacterial pathogen causing a wide range of infections, including pneumonia, sepsis, urinary tract infections, and wound infections. *A*. *baumannii* has emerged as a significant healthcare-associated pathogen due to its high level of antibiotic resistance. The global spread of antibiotic-resistant strains of *A*. *baumannii* has resulted in limited treatment options, leading to increased morbidity and mortality rates, especially in vulnerable populations such as the elderly and immunocompromised individuals, as well as longer hospital stays and higher healthcare costs. Further complicating the situation, multi- and pan-drug-resistant strains of *A*. *baumannii* are becoming increasingly common, and these deadly strains are resistant to all or almost all available antibiotics. *A*. *baumannii* employs various clever strategies to develop antibiotic resistance, including horizontal transfer of resistance genes, overexpression of inherent efflux pumps that remove drugs from the cell, intrinsic mutations, combined with natural selection under antibiotic selective pressure leading to emergence of successful resistance clones. The typical multidrug resistance phenotype of *A*. *baumannii* is, therefore, an orchestrated collimation of all these mechanisms combined with the worldwide spread of “global clones,” rendering infections caused by this pathogen challenging to control and treat. To address the escalating problem of antibiotic resistance in *A*. *baumannii*, there is a need for increased surveillance, strict infection control measures, and the development of new treatment strategies, requiring a concerted effort by healthcare professionals, researchers, and policymakers.

## *Acinetobacter baumannii* is a highly resistant, globally distributed hospital pathogen

*A*. *baumannii* is a gram-negative opportunistic pathogen, and a notorious “ESKAPE” bacteria, the leading cause of antibiotic-resistant nosocomial infections globally [1]. *A*. *baumannii* causes a variety of infections including pneumonia, wound, blood and urinary tract infections and presents a significant global burden, with up to 1.4 million cases reported annually [2]. High levels of antimicrobial resistance (AMR) commonly lead to treatment failure [3], with resistance to last-resort beta-lactam antibiotics such as carbapenems (e.g., imipenem, meropenem) presenting particular concern [4,5]. Indeed, carbapenem-resistant *A*. *baumannii* tops the World Health Organisation’s (WHO) list of pathogens prioritised for the development of new antibiotics [6]. Key players belong to 2 major clones, namely ST1 (known as global or international clone 1, also known as GC1 or IC1, respectively) and ST2 (known as global or international clone 2, namely GC2 or IC2, respectively), which account for the vast majority of outbreaks globally [7–11]. Carbapenem-resistant infection outbreaks have surged since the start of the Coronavirus Disease 2019 (COVID-19) pandemic due to a dramatic increase in the number of hospital and intensive care unit (ICU) admissions [12–15].

Here, we provide a snapshot of how the coalition of intrinsic and acquired resistance mechanisms, combined with the global spread of resistance clones, has resulted in this deadly scourge of multidrug-resistant (MDR) *A*. *baumannii* infections spreading among hospitalised patients. In particular, we discuss unappreciated antibiotic resistance mechanisms (e.g., homologous recombination), highlight their role in resistance gene acquisition, and show how they contribute to the overall resistance and success of *A*. *baumannii*.

## Mobile genetic elements play significant roles in the acquisition and spread of antibiotic resistance genes

**Fig 1:**
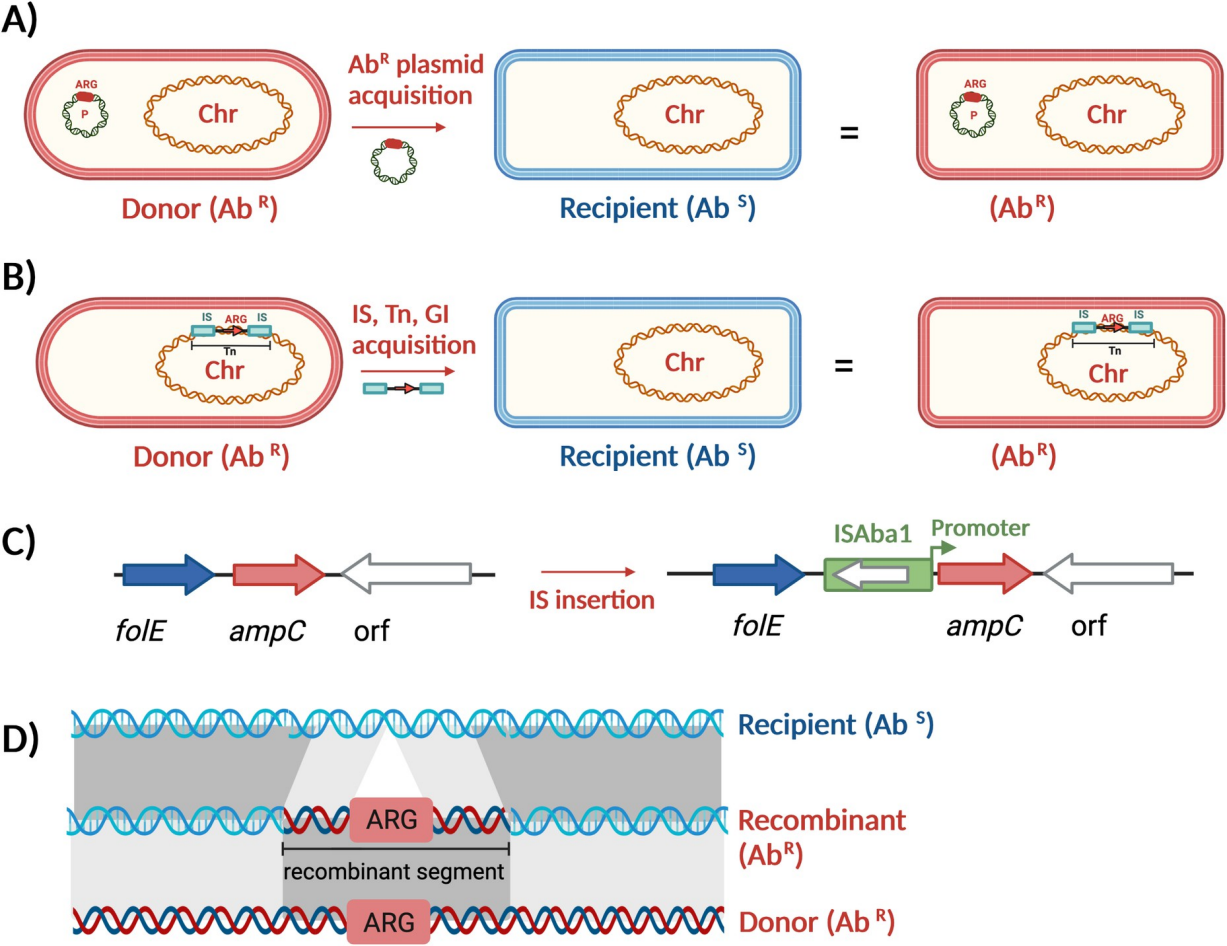
Schematic representation of mechanisms involved in antibiotic resistance of *A*. ***baumannii*.** (**A**) indicates the acquisition of a plasmid that carries antibiotic resistance genes; (**B**) is an schematic of antibiotic resistance gene acquisition via insertion of a composite Tn onto the chromosome (marked “Chr”); (**C**) indicates the insertion of ISAba1 (IS) upstream of the chromosomal *ampC* gene, providing the gene a strong promoter and therefore enhancing expression level leading to resistance to third-generation cephalosporins; (**D**) is a schematic of acquiring a resistance region (GI, T, etc.) via its flanking sequences—of any length—via homologous recombination [25]. ARG, antibiotic resistance gene; GI, genomic island; IS, insertion sequence; Tn, transposon.

In *A*. *baumannii*, the emergence of AMR most often is mediated by the acquisition of antibiotic resistance genes via a wide range of mobile genetic elements (MGEs) including genomic islands (GIs), transposons (Tns), integrons, insertion sequences (ISs), and plasmids [3,4]. Resistance elements (Tn, GI, IS, plasmids, etc.) can insert into chromosomes (Tn, GI, and IS can also insert into plasmids), spreading resistance genes to new *Acinetobacter* cells or be maintained on plasmids in the cells (Fig 1A and 1B).

### Transposons and genomic islands

Several chromosomal GIs and Tns play a leading role in introducing antibiotic resistance genes, including those specific to a given sequence type and others shared between all clones. In ST1 strains, variants of AbaR-type GIs found in the chromosomal *comM* gene carry several heavy metal and antibiotic resistance genes, including those conferring resistance to aminoglycosides [11]. The AbaR-type GIs are made of a backbone Tn and a resistance region in the middle consisting of various complete and incomplete fragments from well-known Tns [16]. In ST2 strains, which are, by far, the most abundant sequence type globally, resistance genes are located on different GI types, namely AbGRI1-5. These GIs contain genes that confer resistance to several antibiotic families, including aminoglycosides, carbapenems, and beta-lactams [17–21]. Like AbaR-type islands in ST1 strains, AbGRI1 variants also consist of a Tn backbone, which is related to the backbone of AbaR-type islands, and occupy the exact chromosomal location as AbaR-type islands [21]. AbGRI2, AbGRI3, and AbGRI4 are also chromosomal GIs made of DNA segments, flanked and formed by IS*26* and several antibiotic aminoglycosides and extended-spectrum ß-lactam resistance genes [17,20]. However, other clones, such as ST79 and ST85 strains, harbour variants of Tn*7*, which play a significant role in the acquisition and spread of diverse antibiotic resistance genes, including for amikacin (*aphA6*) and carbapenem (*bla*_NDM_) [22,23].

In addition to the clone/ST-specific genomic resistance islands, a range of shared composite Tns that are sequence-type agnostic play a crucial role in the acquisition and dissemination of resistance genes across *A*. *baumannii*. For example the small Tns Tn*2006*, Tn*2007*, Tn*2008*, and Tn*2009* mobilising the *oxa23* carbapenem resistance gene [4,9], or AbaR4, the 16-kb GI that carries Tn*2006* (containing *oxa23*), are widely spread across different clones [4,5].

### Plasmids

It is becoming increasingly clear that plasmids are also responsible (in addition to GIs) for shuffling important antibiotic resistance genes around within *A*. *baumannii* [24]. Although previously underappreciated, it has now been established that several (unusual) plasmid types play a significant role in the spread of resistance genes. For example, the small plasmid pRAY (6 kb) and its variants are widely distributed in *Acinetobacter* spp., carry the *aadB* gene, and are the most common cause of resistance to gentamicin and tobramycin in *A*. *baumannii* [25]. Further, conjugative plasmids have facilitated the spread of the *oxa23* (carbapenem) and the *aphA6* (amikacin) resistance genes, using the RP-T1 (formerly Aci6; encoding a Rep belonging to Pfam03090) replication initiation protein and the MPF_F_ conjugation system, respectively [24,26–28]. Other large exotic conjugative plasmids such as those related to pA297-3, pAB3, or pD46-4, which encode the MPF_I_ conjugation system, also carry a wide range of common antibiotic resistance genes, including *oxa23*, *strAB*, and *sul2* [29,30]. Conjugative plasmids related to pA297-3 family have also been attributed to pathogenicity and regulation of virulence determinant (e.g., in urinary tract infections) [31]. Moreover, variants of small plasmid types that encode the Rep_3 replication initiation proteins (Pfam01051) are also crucial in acquiring and disseminating the *oxa24* and *oxa58* carbapenem resistance genes [24,32].

## Homologous recombination is a significant yet overlooked mechanism of resistance acquisition

Homologous recombination (HR) is a type of host-mediated genetic recombination through which segments of DNA (up to hundreds of kb) are exchanged between 2 shorter identical (or very similar) DNA sequences (down to tens of bps) [33,34]. HR plays an important, yet often overlooked, role in bacterial genomes’ evolution and horizontal gene acquisition [33,34]. Recently, the effects of HR on resistance spread in *A*. *baumannii* are becoming elucidated. For example, while resistance to third-generation cephalosporins is known to occur via the insertion of ISAba1 upstream of the intrinsic chromosomal *ampC* gene (Fig 1C), the IS can be acquired either via direct IS insertion, as mediated by classical transposition, or from DNA exchange with a different strain, as mediated by HR between large DNA segments present in several ST1 and ST25 lineages [35–37]. In another example, the AbGRI3 island (approximately 20 kb), which includes several resistance genes (*armA*, *aphA1*, *msr-mph*(E), *sul1*, *aadA1*, *and catB8*) and is common in ST2 strains, is acquired from another ST2 (GC2) via HR, as part of a larger (>56 kb) DNA chromosomal segment by ST1 strains [37]. HR has been shown to be responsible for a non-ST2 *A*. *baumannii* hospital isolate acquiring a genomic resistance island (AbGRI5, carrying the *armA*, *msr*-*mph*(E), *sul1*, *bla*_PER-1_, *aadA1*, *cmlA1*, *aadA2*, *bla*_CARB-2,_ and *ere(B)* resistance genes) from ST2 (GC2) [18]. Finally, it has been shown that the classic *gyrA* and *parC* mutations that lead to fluoroquinolone resistance are freely exchanged throughout *A*. *baumannii* isolates by acquiring DNA segments containing the mutated alleles, via HR [37]. Although these examples highlight some impacts of HR on the acquisition and evolution of resistance genes, further investigations across the broad range of sequence types and geographical areas are needed to determine the full, potentially large, role of HR in *A*. *baumannii* resistance.

## Intrinsic mechanisms of antibiotic resistance

**Fig 2:**
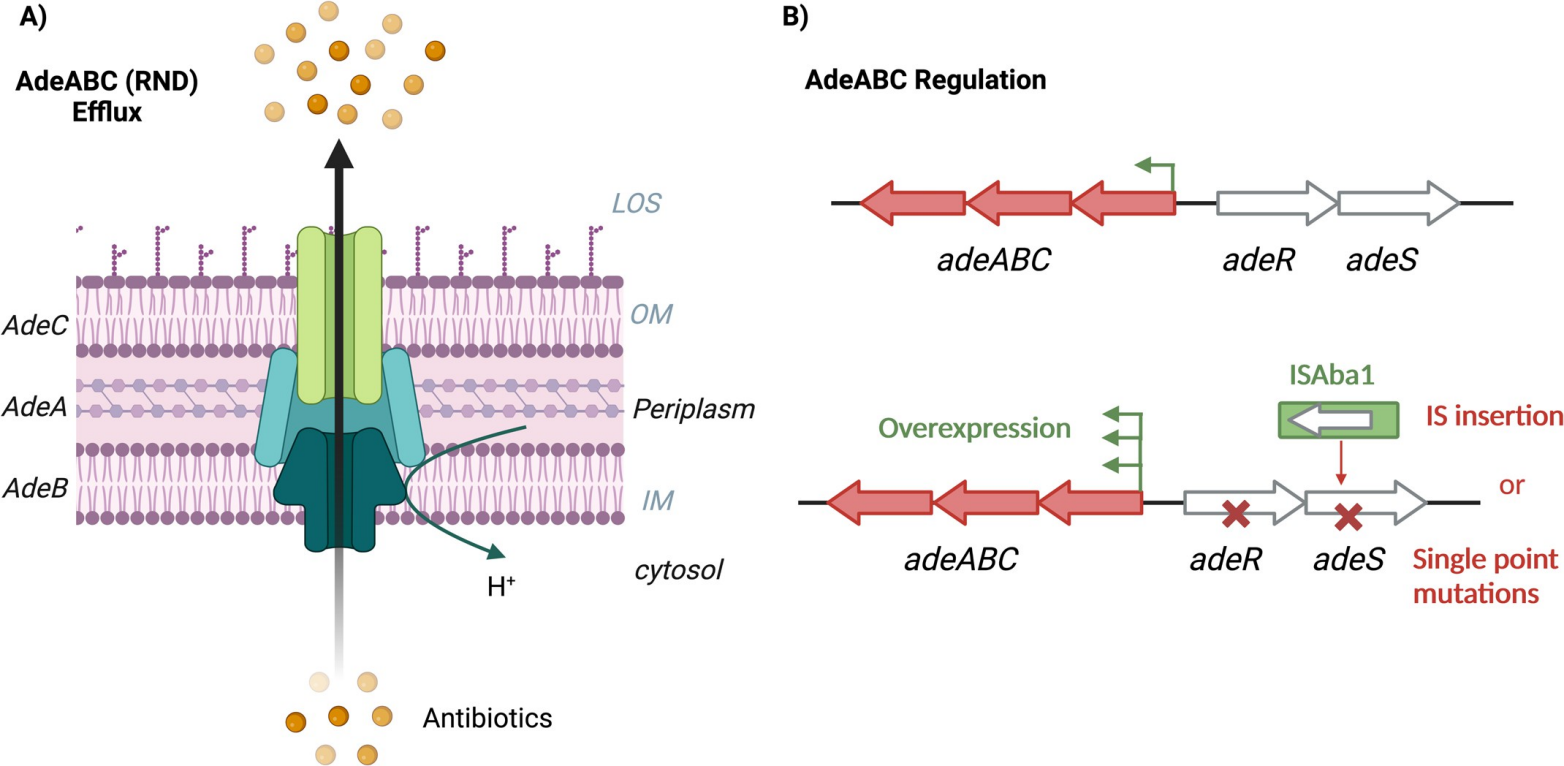
Intrinsic resistance mechanisms. (**A**) AdeABC pump (of the RND family) is the most important for efflux-mediated drug resistance in *A*. *baumannii*. (**B**) Regulation of AdeABC is mediated by AdeRS, where ISs and single point mutations can disrupt these genes and result in overexpression. IM, inner membrane; IS, insertion sequence; LOS, lipooligosaccaride; OM, outer membrane; RND, resistance-nodulation-division.

One resistance mechanism classically associated with *A*. *baumannii* is their impressive suite of efflux transporters, which actively pump antibiotics and biocides out of the periplasm via proton motive force before cell damage occurs. There are 5 major classes of efflux pump families associated with resistance in *A*. *baumannii*, namely, resistance-nodulation-division (RND; e.g., AdeABC), major facilitator superfamily (MFS; e.g., TetA, AmvA), multidrug and toxic efflux (MATE; e.g., AbeM), the small multidrug resistance (SMR; e.g., AbeS), and ATP binding cassette (ABC; e.g., MacAB/TolC). The most significant resistance-associated pumps in *A*. *baumannii* are part of the RND family efflux pumps, which have broad specificity and are present in most strains [38]. The most common resistance pumps are encoded by *adeABC*, *adeFGH*, and *adeIJK* [39,40]. In addition to the presence and absence of these efflux pumps, the regulation of their activity can dictate resistance; some are constitutively expressed, and some are tightly regulated and activated in response to specific drugs or environmental cues. For example, the *adeABC* operon encodes AdeA (membrane fusion protein), AdeB (multidrug transporter), and AdeC (outer membrane protein) to form a pump spanning the inner and outer membrane (Fig 2A), and its expression is regulated by the AdeRS TCS in response to fluoroquinolone exposure (Fig 2) [41]. However, constitutive overexpression of *adeABC* results in broader resistance (to aminoglycosides, tetracyclines, chloramphenicol, β-lactams, and tigecycline) and can occur as a result of ISAba1 insertion upstream of *adeABC* (Fig 2B) or via point mutations in *adeR* or *adeS*, having major clinical implications [42–44]. Interestingly, inherent efflux activity seems to act in a strain-dependent context, where certain pumps are expressed in different strains; for example, AdeABC appears to be more active in ATCC17978, whereas AdeIJK is more active in AB5075 (GC1) for drug resistance [40,45].

However, other intrinsic resistance mechanisms are employed by the *A*. *baumannii* cells to evade antibiotic action, including target site modification via single point mutations, where the active site of the drug is modified to render it ineffective [46] or IS insertion to change regulation as is seen across several antibiotic classes.

## Resistance to colistin and other polymyxins

Colistin and other polymyxins are a last resort within our arsenal of antibiotics that largely remained effective against MDR *A*. *baumannii*. However, increasing levels of resistance are being recorded, and several molecular drivers have been identified that underpin this last-line resistance. Colistin resistance is due to point mutations in key genes that cause the bacterium to alter the sugar moieties of or even completely shed its lipooligosaccaride (LOS), resulting in the colistin being unable to bind to its target [47,48]. In the case of LOS loss, this was found to be due to ISAba11 disrupting the Lipid A biosynthesis genes *lpxA/C*. Interestingly, the loss of LOS seems to be an *A*. *baumannii*-specific resistance strategy, as other gram-negative bacteria never lose their lipopolysaccharide (LPS), but only use Lipid A modification. Other mutations target specific downstream effectors that affect lipid and membrane production and stability, including *lpxA* [49] and the *mla* operon [50].

Colistin resistance mutations arise in two-component systems (TCSs) that control the production and modification of LOS, namely in PhoPQ, PmrAB [51], BaeSR [50], or StkSR [52]. For example, *pmrA/B* TCS controls *pmrC* expression, which encodes a pEtN transferase to modify the Lipid A, providing resistance. So far, 68 amino acid changes have been identified for *A*. *baumannii* across this system that mediates colistin resistance [53], and the mutations only need to be present in a subset of the population, presenting as colistin heteroresistance [54].

Recently, colistin resistance has also been described as been carried on plasmids, via the *mcr* gene, and this has been demonstrated for *A*. *baumannii* from clinical isolates and even environmental samples [55].

## Resistance to carbapenems and third-generation cephalosporins

Unlike most gram-negative bacteria (e.g., Enterobacterales), in *A*. *baumannii*, resistance to carbapenems is due to the acquisition of carbapenem-hydrolysing oxacillinase-encoding (class D) genes such as *oxa23*, *oxa24*, and *oxa58* [4]. Of these, *oxa23* is by far the most abundant in many countries, while *oxa24* and *oxa58* appear to be more dominant in specific regions. The *oxa23* gene moved by Tn*2006*-9, with Tn*2006* being the most abundant Tn that spreads this gene [4]. Other carbapenem resistance genes such as metallo-ß-lactam resistance genes are also reported but remain rarely seen [4].

Genes encoding the extended-spectrum beta-lactamases (ESBLs) are responsible for resistance to extended-spectrum beta-lactam antibiotics in most gram-negative bacteria. However, in *A*. *baumannii*, it occurs via insertion if an ISAba1 (or ISAba125) upstream of the chromosomal *ampC* gene, which provides the gene with a strong promoter enhancing the suppression level and, therefore, high levels of resistance to third-generation cephalosporins, as well as resistance to beta-lactam inhibitors, like sulbactam [56]. This occurs either via the classic insertion of ISAba1 upstream of the chromosomal *ampC* gene or as indicated above, by an acquisition of an exogenous DNA segment containing an ISAba1-activated *ampC* gene via homologous recombination replacing large segments of the chromosome [36].

## Resistance to aminoglycosides and fluoroquinolones

Aminoglycoside resistance occurs by the acquisition of genes encoding different families of aminoglycoside modifying enzymes often carried by composite Tns (e.g., Tn*6020* carrying the *aphA1* kanamycin, neomycin, and gentamicin resistance gene) or gene cassettes (e.g., the *aacC1* gentamicin resistance gene found on class 1 integrons) in major global clones (ST1 and ST2) [16,57].

Fluoroquinolone resistance is mainly due to the generation of point mutations in the active sites of the *gyrA* and *parC* genes that encode the cell’s DNA gyrase and topoisomerase IV enzymes, which are required for cell replication and survival. Mutations often occur by fluoroquinolone selective pressure. However, mutations can also be obtained via the acquisition of DNA segments—that include the mutations—by HR from an exogenous source (a strain that belongs to a different ST) [37].

## Complex evolutionary pathways lead to multiple antibiotic resistance gene acquisitions combined with the global spread of resistant clones

The literature on antibiotic resistance of *A*. *baumannii* commonly highlights that *A*. *baumannii* has an incredible ability to develop resistance to many antibiotics. Although true, a major driver of resistance is also due to the clonal global expansion of successful resistant strain. Notably, while new resistant clones are emerging and isolates are continually gaining resistance genes, the bulk of the global MDR *A*. *baumannii* infections are still due to a few successful clones that are disseminated globally, typically those belonging to global clones GC1 (represented by ST1) and GC2 (represented by ST2) and a few additional sequence types, e.g., ST10, ST15, ST79, and ST85 [4]. Those belonging to ST15, ST25, ST79, and ST85 are important and most prevalent in certain geographical regions (e.g., ST85 in the Middle East or ST79 in South America) [4,58–64]. It is important to note that multidrug resistance is often the result of the accumulation of a characteristic suite of antibiotic resistance genes for each clone, which then spreads. For example, clone-specific GIs often carry multiple resistance genes and insert into the chromosomes rendering cells resistant to several antibiotics in a single event. In contrast, nonspecific (shared) resistance elements (Tn, GI, plasmids, etc.) can be incorporated into both the chromosomes and plasmids and help accumulate the resistance genes leading to a complex picture. For example, it has been shown that AbaR-type islands (with several resistance genes) entered a single member of global clone 1 (GC1) in the mid-1970s (before it was globally distributed) and continued to evolve in situ forming many variants [16]. Thus, at least in ST1 strains, multi-, extensive-, and pan-drug resistance is the result of many acquisition/deletion events initiated by the acquisition of GIs, and other resistance determinants via homologous recombination providing the huge selective advantage to spread globally, followed by additional decorations of subsequent Tns or plasmids that carry carbapenem resistance genes. Several other globally distributed sequence types (ST2, ST25, ST79, and ST85) have also achieved multiply-extensive and pan-drug resistance phenotypes through completely different evolutionary paths. However, they share a common feature in which gene acquisition, either via MGEs or homologous recombination, and single base mutations, or mutation acquisition via homologous recombination, play a pivotal role in driving the resistance phenomenon.

It should be emphasised that in addition to numerous clever intrinsic and acquired mechanisms, the global spread of few highly successful clones and their lineages drive *A*. *baumannii* into becoming the globally successful and near-impossible-to-treat pathogen than it is today.

Together with the current lack of new antibiotics to treat resistant strains combined with the uncertainty about the discovery and effectiveness of new antibiotics, improved infection control policies, increased surveillance, and new treatment strategies are urgently needed. This requires a concerted global effort by researchers, healthcare professionals, policymakers, and governments.
